# Information-rich localization microscopy through machine learning

**DOI:** 10.1038/s41467-019-10036-z

**Published:** 2019-04-30

**Authors:** Taehwan Kim, Seonah Moon, Ke Xu

**Affiliations:** 1 Department of Electrical Engineering and Computer Sciences, University of California, Berkeley, CA 94720 USA; 2 Department of Chemistry, University of California, Berkeley, CA 94720 USA; 3 Chan Zuckerberg Biohub, San Francisco, CA 94158 USA

**Keywords:** Image processing, Machine learning, Imaging techniques, Super-resolution microscopy

## Abstract

Recent years have witnessed the development of single-molecule localization microscopy as a generic tool for sampling diverse biologically relevant information at the super-resolution level. While current approaches often rely on the target-specific alteration of the point spread function to encode the multidimensional contents of single fluorophores, the details of the point spread function in an unmodified microscope already contain rich information. Here we introduce a data-driven approach in which artificial neural networks are trained to make a direct link between an experimental point spread function image and its underlying, multidimensional parameters, and compare results with alternative approaches based on maximum likelihood estimation. To demonstrate this concept in real systems, we decipher in fixed cells both the colors and the axial positions of single molecules in regular localization microscopy data.

## Abstract (web summary)

Single-molecule methods often rely on point spread functions that are tailored to interpret specific information. Here the authors use a neural network to extract complex PSF information from experimental images, and demonstrate this by classifying color and axial positions of emitters.

## Introduction

Originally developed toward the specific goal of superior spatial resolution, single-molecule localization (super-resolution) microscopy (SMLM), including STORM/(F)PALM^1–3^ and PAINT^4^, has evolved in recent years into a generic tool for sampling diverse biologically relevant information at the nanoscale^5^. For example, when combined with environment-sensitive dyes, intracellular heterogeneity in the local chemical environment can be mapped by concurrently obtaining the spatial positions and emission spectra of millions of single fluorescent molecules^6^. Consequently, the full potential of SMLM is expected to be unleashed through the proper multidimensional analysis of the emission wavelength^5^, brightness, dipole orientation^7^, as well as the axial (3D) position^8^ of single molecules.

To date, to extract information beyond the in-plane location, e.g., the emission wavelength and the axial position, of single emitters, would often oblige the explicit encoding of such high-dimensionality information into the diffraction pattern of single molecules (point spread functions; PSFs) through optical aberrations and alterations^5,8^, including astigmatism^9^, interference^10^, wavelength-dependent splitting^11^, dispersion^12^, and wave-front modification^13^. The resultant engineered PSF shape and intensity then help establish best-fit models between experimental observables and fluorophore characteristics. Such approaches, each often optimized for a single parameter of interest, inevitably increase the PSF size and/or necessitate the splitting of fluorescence across different channels, and so often incur complicated optics and compromised performances between different parameters. While recent work^14,15^ has studied the PSF design for the simultaneous estimation of color and axial position, added optics and enlarged PSFs are still involved, and proper calibration of such Fourier optics-heavy systems is challenging^16^.

Even the simplest PSF obtained from an unmodified microscope is rich in information—in addition to the axial location embedded in the defocused PSF, which has been examined in recent work^17,18^, the emission wavelength of a fluorophore also sets the scale of PSF in all three dimensions^19^. Contributions from the two sources are distinct yet subtle and would be hard to decouple via simple models given the difficulties in fully characterizing all system-specific properties. Although recent work^18,20^ leveraging spline models may help account for the subtleties in realistic PSF images and thus potentially decipher this extra information, the construction of such models usually requires reference PSF stacks acquired under ideal condition, e.g., bright fluorescent beads of precisely determined 3D positions and emission wavelength.

In this work, we present a data-driven approach in which the relationship between a PSF image, obtained from an unmodified commercial microscope, and the underlying multidimensional characteristics of an emitter is directly established by a supervised machine learning algorithm. A related approach has been recently used in astronomy for stellar classification^21^. Although SMLM faces additional challenges associated with the vast range of axial positions (as opposed to stars always at infinity), it benefits from the ready access to arbitrary amounts of experimental PSFs that may be acquired under identical conditions, which has motivated emerging work that leveraged machine learning for single-particle 3D localization^22,23^ and color separation^24^. By training generic learning models using such datasets, an end-to-end framework from raw, noisy PSF images to the molecule characteristics can be constructed.

## Results

### Construction of color-separating and axial-localization ANNs

**Fig. 1:**
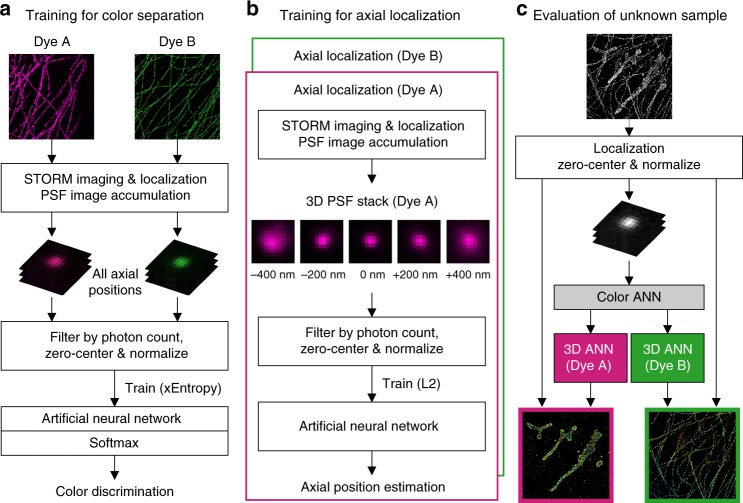
Workflow of the machine learning-based multidimensional SMLM. **a** A color-separating ANN is trained using samples each singly labeled by one known fluorophore, in which PSFs at different axial positions are well-represented. **b** ANNs for resolving the axial position are separately trained for each fluorophore using PSFs of known axial positions. **c** For the analysis of unknown samples, single-molecule images are localized in 2D, and first fed into the color-separating ANN described in **a**. The color-separated single-molecule images are then separately fed into the axial-localization ANNs trained with the corresponding fluorophores, as described in **b**. The resultant color and axial position information are then combined with the 2D localization of each molecule to generate the final multidimensional SMLM data

To demonstrate this concept, we developed a method for machine learning-based 3D multi-color SMLM (Fig. 1 and Methods). With typical experimental pixel sizes ( ~ 100 nm), the dimensionality of the PSF images is moderate (modeled as 13 × 13 pixels), and thus artificial neural networks (ANN) with multiple hidden layers^25^ were directly used as our learning model. ANN is beneficial here as it possesses excellent representational power, with no requirement of domain-specific knowledge on the input data to construct nonlinear models. Moreover, as long as a sufficient amount of input training data is provided to the ANN, noise in the data averages out during training process given proper regularization, and ANN eventually manages to extract underlying structures^26^. Consequently, it is well-suited for the limited photon budget and heavy pixelation in SMLM. Finally, ANN training only requires the ground truth of the parameter of interest. Namely, it gradually establishes the relationship between the raw input and the inference target (e.g., color or axial position) in a flexible, end-to-end fashion while being insensitive to other parameters (e.g., *x*/*y* position). In contrast, for approaches in which parametric models are constructed by fitting to experimentally obtained PSF images^20,27^, experimental images always need to be tied to precisely determined 3D positions. Reference PSFs are thus usually acquired using bright fluorescence beads, which may not accurately represent the PSFs of single molecules in SMLM experiments.

One ANN with a final Softmax layer was first trained using cross-entropy loss to determine the emitter color of each PSF. Once trained, the final Softmax output provided an estimate for the conditional probability distribution of the fluorophore color, which enabled the classification of each PSF image with known confidence (Fig. 1a and Methods). For this color-separating ANN, training data for different fluorophores were separately prepared from multiple imaging sessions performed under the same experimental conditions as the final sample, but using only one known fluorophore at a time. The training data contained sufficient samples for fluorophores at different axial positions within the depth of field ( ~ ± 500 nm of the focal plane), so that the ANN was trained to recognize fluorophores for all axial positions.

In parallel, ANNs for resolving the axial position of the emitter were separately trained for each fluorophore using L2 loss so that the final output was a scalar value^28^ corresponding to the decoded axial position (Fig. 1b). Training data for these axial-localization ANNs were collected by step-scanning samples each containing one specific fluorophore, as is typically performed for the calibration in existing 3D SMLM methods^9^.

Once both trainings were completed, SMLM data from unknown samples were localized in 2D, and the single-molecule images were first fed into the above color-separating ANN (Fig. 1c). The resultant, color-separated single-molecule images were then separately fed into the above axial-localization ANNs trained with the corresponding fluorophores (Fig. 1c). Multidimensional SMLM data were thus obtained by integrating the ANN-inferred color and axial information with the initial 2D-localization results.

### Performance of the color-separating ANN

**Fig. 2:**
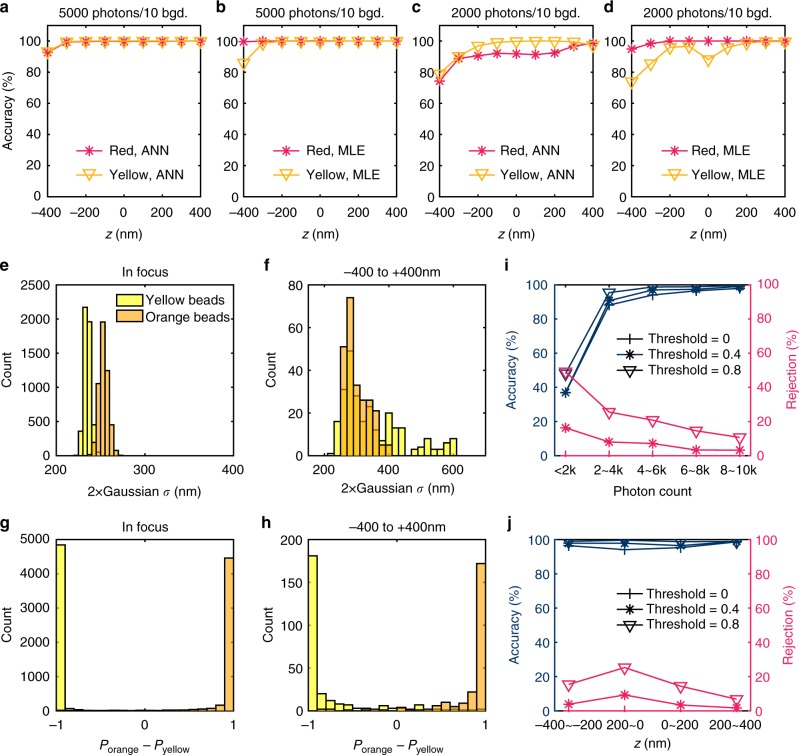
Performance of the color-separating ANN. **a**–**d** Comparison of the color-separation performance of the ANN and cubic-spline MLE for simulated PSFs of yellow (600 nm) and red (700 nm) point sources with 5000 **a**, **b** and 2000 **c**, **d** emitted photons and a background of 10 photons/pixel. **e**, **f** PSF size (2*σ* of 2D Gaussian fitting) distributions of experimental images of yellow and orange beads (with emission peaks at 515 and 560 nm, respectively) emitting ~ 4000 photons, when the beads are at the focal plane **e** and as the focus is uniformly scanned over ± 400 nm **f**. **g**, **h** Outputs of the color-separating ANN for the same PSFs in **e**, **f**, presented as the distribution for the differences in the evaluated probabilities of each bead being orange vs. being yellow. **i**, **j** Accuracy of classification (left axes) and rejection rate (right axes) in the presence of defocusing, as a function of photon count for all z-positions **i** and as a function of z position for beads brighter than 4000 photons **j**, for confidence thresholds of 0, 0.4, and 0.8

We first examined the performance of the color-separating ANN using simulated yellow (600 nm wavelength) and red (700 nm wavelength) PSFs that account for index discontinuity in the sample area^19^ (Supplementary Fig. 1). For comparison, we also modeled the PSF with cubic splines^20^, and determined color through maximum likelihood estimation (MLE) by minimizing the likelihood error in the MLE fitting to the PSF stacks of the two different emission wavelengths. For both training the neural networks and the construction of the cubic-spline model, we used a PSF reference stack of 10000 simulated photons over ± 600 nm axial (*z*) range in 20 nm steps. For the analysis of unknown PSFs, the PSF was either directly fed to the trained neural networks, or for MLE, fitting was performed twice for each color with negative and positive initial *z* values, respectively, so that the result with a lower likelihood error was selected to overcome the limitation of MLE being sensitive to initial parameters^18^. At a fixed simulated background of 10 photons/pixel, we found that at 5000 simulated photons, both ANN and MLE achieved near-perfect color separation (Fig. 2a, b). At 2000 simulated photons (Fig. 2c, d), ANN slightly outperformed MLE for the yellow PSFs, especially for *z* = 0 nm, whereas MLE performed better for the red PSFs.

For experiments, we used two types of 40-nm dia. fluorescent beads that differed by 45 nm in emission wavelength (yellow and orange), and images were acquired over ± 400 nm around the focal plane in 50 nm steps (Methods and Supplementary Fig. 2). When the beads were at the focal plane, fitting to simple Gaussian models yielded PSF sizes (2*σ*) that were directly proportional to the emission wavelength, as expected, and this difference gave adequate separation of the two colors (Fig. 2e). However, this separation quickly fell apart when results from different axial positions were mixed: unsurprisingly, defocusing led to substantially increased PSF sizes, and so this parameter no longer offers useable color separation (Fig. 2f).

In contrast, our color-separating ANN recognized the nuances in the PSF patterns due to differences in color vs. differences in axial position, and thus offered excellent color separation both in the absence and presence ( ± 400 nm range) of defocusing (Fig. 2g, h). As mentioned, the output of this ANN gives the conditional probabilities of each given single-molecule image being classified as certain types of fluorophores. In the binary yellow-orange system, the results can be simplified as the difference *Δ* between the evaluated probabilities of being orange and being yellow for every image (Fig. 2g, h). Even in the presence of defocusing, simple classification based on *Δ* > 0 and *Δ* < 0 gave excellent identification for beads brighter than 4000 photons (Fig. 2h, i), with little dependence on the axial position within the ± 400 nm focal range (Fig. 2j). Note in STORM experiments, an average of > 5000 photons is often obtainable for single molecules^9,29^. Reducing the photon count to the range of 2000-4000 photons led to a decrease in accuracy to 88% (Fig. 2i), but this result was improved to 95.4%, by only keeping classifications with |*Δ*| above the confidence threshold of 0.8, at the expense of rejecting 25% classifications (Fig. 2i). Our ANN approach can thus be tuned for experiments that emphasize color-separation accuracy vs. experiments that emphasize the retention of molecules.

### Performance of the axial-localization ANN

**Fig. 3:**
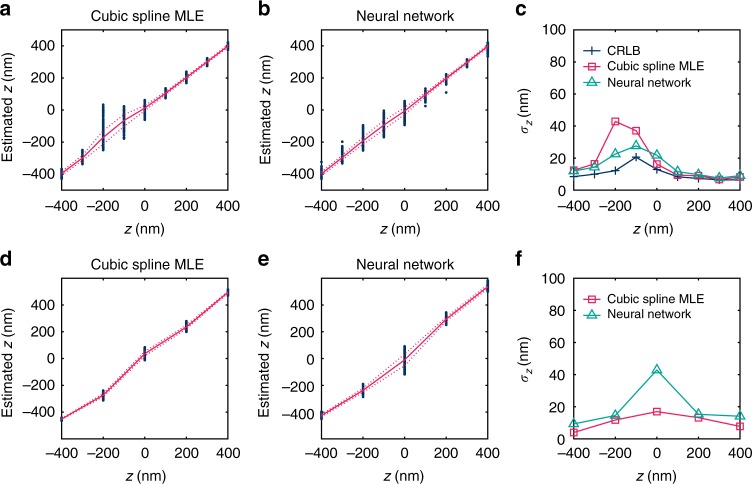
Comparison of the results of the axial-localization ANN with cubic-spline MLE and with the Cramer-Rao lower bound. **a**, **b** Estimated *z* positions vs. the ground truth over ± 400 nm of the focal center for simulated point sources of 700 nm wavelength at a brightness of 5000 photons, using **a** MLE with a cubic-spline model and **b** ANN estimation. The scattered data points represent estimated *z* positions at each true *z* position, and the red solid and dash lines give the corresponding mean and standard deviation. **c** *z* precision from **a**, **b**, compared with the Cramer–Rao lower bound calculated from the cubic-spline model. **d**–**f** Corresponding results on experimental images of red fluorescent beads (typical photon count: ~ 15,000)

We next characterized the axial-localization ANN and compared with MLE results based on cubic-spline PSF models. Results on simulated PSFs of a 700 nm wavelength emitter (Fig. 3a–c) showed that both the ANN and cubic-spline MLE results generally followed the trend of the Cramer-Rao lower bound (CRLB), although a somewhat deteriorated performance was found at *z* = −200 nm for the particular MLE fitter we used. For experimental PSFs acquired with bright fluorescent beads, ANN generally achieved comparable results as cubic-spline MLE but showed a lower performance for *z* = 0 nm (Fig. 3d–f).

Together, our results showed that ANN achieves good color separation and axial localization for unmodified PSFs, and its performance is generally comparable to the state-of-the-art parametric PSF models. However, for experimental implementation, the construction of ideal parametric PSF models relies on ideally measured PSFs, like those obtained above from bright beads, for which the 3D positions of each PSF can be precisely determined. In comparison, ANN should readily extract the underlying structures from a large number of non-ideal PSFs of unknown positions, like single-molecule data from SMLM experiments of cell samples.

### Application to SMLM of cells

To test this possibility, we immunolabeled the microtubules and the outer membrane of mitochondria in adherent COS-7 cells with two STORM dyes, CF568 and Alexa Fluor 647 (AF647). Both dyes were excited within the same STORM imaging session, and resultant single-molecule fluorescence was collected in one single optical path after a multi-notch filter. For training of the color-separating ANN, COS-7 cells singly-labeled by CF568 and AF647 for microtubules were STORM-imaged on the same setup, which naturally contained single molecules at all possible axial positions within the depth of field. For training of the axial-localization ANN, dye-labeled antibodies were attached to the coverslip for step-scanning in the axial direction (Methods).

**Fig. 4:**
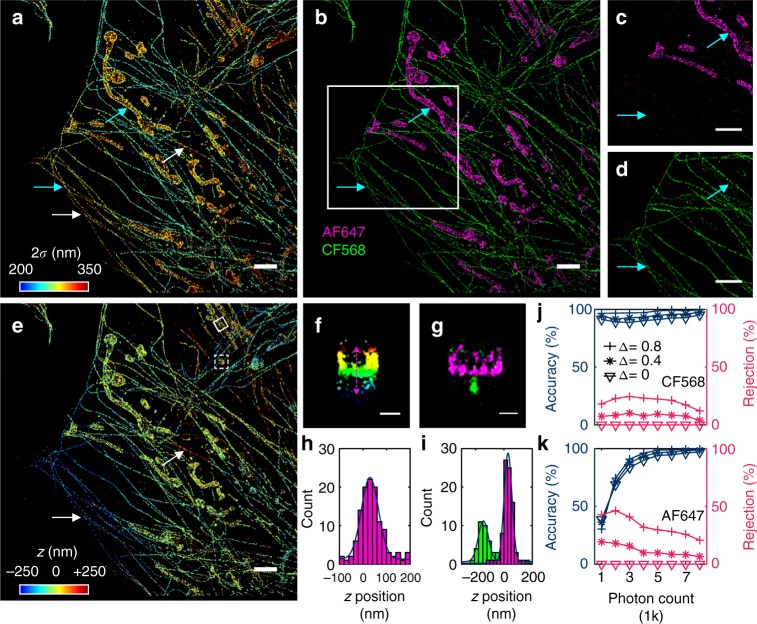
ANN-resolved multicolor 3D SMLM in cells based on unmodified PSFs. **a** STORM image of CF568-labeled microtubules and AF647-labeled mitochondrial outer membrane in a fixed COS-7 cell, colored by the fitted Gaussian width (2*σ*) of the PSF of each molecule, for molecules brighter than 3000 photons. **b** Result of the color-separating ANN for the same dataset, at a confidence threshold of 0.8. **c**, **d** The separated AF647 **c** and CF568 **d** channels for the boxed area in **b**. Cyan arrows in **a**–**d** point to two regions where molecules of similar PSF widths are correctly determined as different colors by the ANN. **e** The merged 3D STORM image after separately determining the axial position of every single molecule based on ANNs respectively trained for AF647 and CF568. Color here presents the axial position (*z*), with blue being closest to the substrate and red being the farthest away. White arrows in **a** and **e** point to two regions of the CF568 microtubule labeling that showed similar defocusing effects but determined by ANN as being on opposite sides of the focal plane. **f**, **g** Vertical cross-sectional views for the **f** solid boxed area (colored by *z*) and **g** dashed boxed area (color separated by the ANN) in **e**. **h** Histogram of the axial (*z*) position along the magenta line in f (*σ* of the fitted Gaussian: 36 nm). **i** Histogram of the axial (*z*) position of the microtubule and the bottom membrane of the mitochondrion in g (*σ* of the fitted Gaussian: 36 and 28 nm, respectively). **j**, **k** Classification accuracy (left axis) and rejection rate (right axis) of the color-separating ANN as a function of photon count, for cells singly labeled by CF568 **j** and AF647 **k**, at confidence thresholds of 0, 0.4, and 0.8. Scale bars, 2 μm **a**–**e**, 200 nm **f**, **g**

Figure 4a presents the acquired STORM image colored by the fitted Gaussian width (2*σ*) of the PSF of each molecule. Here a brightness threshold of 3000 photons was applied, and ~ 40% of the identified single molecules met this criterion. This rejection of dimmer molecules may be compensated by collecting more (currently 20,000) frames of raw STORM data. Whereas it is clear that all the narrowest widths belonged to microtubules, which were stained by the shorter-wavelength dye CF568, larger widths were found at both microtubules and mitochondria (e.g., cyan arrows in Fig. 4a). This result is similar to what we saw in the bead data (Fig. 2f): defocusing broadens the PSF width, and so this simple parameter can no longer be used to separate colors. Remarkably, our color-separating ANN achieved excellent color separation for the entire image independent of axial position (and thus defocusing) (Fig. 4b–d), and consistent results were obtained on different cells over repeated experiments (Supplementary Fig. 3). Quantification of color classification accuracy, as separately determined using fixed cells singly labeled by CF568 (Fig. 4j) and AF647 (Fig. 4k), indicated that at ~ 5000 photons, excellent accuracies of 98.2% were achieved for both dyes at the confidence threshold of 0.8. At ~ 3000 photons, the accuracy for CF568 did not vary noticeably (Fig. 4j), whereas the accuracy for AF647 dropped to ~ 90.4% (Fig. 4k). Lowering the confidence thresholds led to accuracy drops by a few percentage points (Fig. 4j, k). Previous work^29^ has shown that for dyes in these two color channels, through traditional sequential imaging using different optical filter sets, a ~ 8% crosstalk occurred from the 561-nm excited dye into the 647-nm excited dye, whereas crosstalk in the opposite direction was ~ 1%. Our accuracies thus appear to outperform at ~ 5000 photons, a value often obtained in STORM experiments^9,29^. Moreover, in our case, all data were collected within the same optical path in a single STORM session, so we avoided the major difficulties in aligning images from different filter sets.

Based on our successful color classification, two axial-localization ANNs, each trained for AF647 and CF568, were next used to separately decode the axial positions of the molecules in the two color channels, the results of which were recombined into one image for presentation (Fig. 4e and Supplementary Fig. 3). This showed the expected result that the cell edges, thinner in height, were dominated by small *z* values, whereas for regions far away for the cell edges, the cells became thicker and had increased *z* values. White arrows in Fig. 4a, e further point to regions of microtubules, labeled by the same CF568 dye, where similarly increased PSF widths were noted, but the ANN correctly identified one being below the focal plane whereas the other above. Vertical cross-sectional views of the data correctly showed the hollow structure of the mitochondrial outer membrane (Fig. 4f, g), and distributions of the *z* positions for the AF647-labeled mitochondrial outer membrane and the CF568-labeled microtubule both showed standard deviations of 30–40 nm (Fig. 4h, i). We further note that as the two color channels are successfully separated, they may also be separately fed into other recent methods that extract axial positions from unmodified PSFs^17,18^.

## Discussion

Our finding highlights the rich, multidimensional information concealed in the details of the diffraction-limited image of a fluorophore, which was unleashed in this work through machine learning algorithms. Not having to modify the PSF shape or divide single-molecule fluorescence between different optical paths, or to image sequentially, not only simplify experimental implementation, but more importantly, preclude the deterioration in SMLM performance due to enlarged PSFs and/or split channels, as well as the need to align localizations from different channels. Moreover, once trained, evaluation was straightforward and fast ( > 3.3 × 10^5^ molecules/s with GPU acceleration) for both the color-separating and the axial-localization ANNs.

One limitation of our current work is good training samples for the *z* position. In the presence of index mismatch and supercritical angle fluorescence, PSFs acquired from coverslip-attached single molecules would be different from those labeled inside cells. While this is a common challenge for 3D-SMLM, recent work has shown the possibility to overcome such limits through imaging single molecules attached to known structures such as microspheres^30^, as well as fluorescent beads encapsulated in an agarose gel^18^. Incorporating such approaches would help improve *z* precision.

Finally, we note that our end-to-end framework may be further extended to determine more parameters. As a first step, we evaluated the performance of lateral position (*x*/*y*) estimation using ANNs with simulated PSF images and achieved good precision when compared to the CRLB (Supplementary Fig. 4). The difficulty of applying such ANN analysis for lateral positions, as well as for other possible parameters, including the signal and background levels, however, resides with the difficulty in constructing good training sets with known *ground truth*. Together, we expect the co-evolution of our data-driven end-to-end framework with ongoing efforts on PSF engineering^8^ should lead to new improvements, and conceivably, new types of imaging modalities, for multidimensional SMLM.

## Methods

### Optical setup

STORM and bead experiments were performed on a Nikon Ti-E inverted fluorescence microscope using an oil-immersion objective lens (Nikon CFI Plan Apochromat λ 100 × , NA 1.45) and the native 1.5x magnification on the microscope, without any modifications to the imaging path. Lasers emitting at 644, 561, and 488 nm were introduced to the back focal plane of the objective lens via a multi-line dichroic mirror (ZT405/488/561/640rpc-uf2, Chroma). A translation stage shifted the laser beams toward the edge of the objective lens so that they entered slightly below the critical angle, illuminating < 1 µm into the sample. Emission was filtered by a multi-notch filter (ZET405/488/561/640 m, Chroma) and recorded by an EM-CCD camera (iXon Ultra 897, Andor). Effective magnification and pixel size were ~ 150x and ~ 107 nm, respectively.

### Bead samples

For bead experiments for color-classification (for both the training of the ANNs and the analysis of unknown samples), 40 nm dia. fluorescent beads from Invitrogen (F10720; yellow and orange FluoSpheres with emission peaks at 515 and 560 nm, respectively) were diluted in Dulbecco’s phosphate buffered saline (DPBS), mixed, and sealed between a glass slide and a pre-cleaned #1.5 thickness coverslip, and imaged with the above optical setup. The 488-nm and 561-nm lasers were used to excite the two types of beads to similar levels of brightness. To record images at different axial positions, the objective lens was scanned by the built-in motor over a range of −400 to + 400 nm of the focal plane in 50 nm steps. To compare the performance of the axial-localization ANN with cubic-spline MLE, 40 nm dia. fluorescent beads from Invitrogen (F8789; dark red FluoSphere with an emission peak at 680 nm) were similarly prepared as described above, excited with the 644-nm laser, and scanned from -400 nm to + 400 nm in 50 nm steps.

### Cell samples

For STORM experiments (training of the color-separating ANN and the ANN analysis of unknown samples), COS-7 cells were plated on #1.5 coverslips to reach a confluency of ~ 50% in ~ 1.5 days, and fixed with 0.1% glutaraldehyde and 3% paraformaldehyde in DPBS at room temperature. The sample was quenched with 0.1% sodium borohydride in DPBS and rinsed with DBPS three times. Primary and secondary antibodies were diluted in a blocking buffer (3% BSA + 0.1% Triton X-100 in DPBS) and labeled as described previously^9^. Primary antibodies were mouse anti-tubulin (Abcam ab7291) for microtubules and rabbit anti-Tom20 (Santa Cruz sc-11415) for mitochondrial outer membrane. Secondary antibodies were AF647-labeled goat anti-mouse IgG1 (Invitrogen A21240), AF647-labeled goat anti-rabbit IgG (Invitrogen A21245), and donkey anti-mouse IgG (Jackson ImmunoResearch) conjugated with a CF568 succinimidyl ester (Biotium 92131). Samples for training the color-separating ANN were singly labeled for microtubules with CF568 or AF647, whereas for the two-color unknown samples, microtubules and the mitochondrial outer membrane were respectively labeled with CF568 and AF647. The sample was mounted in a STORM buffer [10% (w/v) glucose, 120 mM cysteamine, 0.8 mg/mL glucose oxidase, and 40 µg/mL catalase in Tris-HCl (pH 7.5)] and imaged using the optical setup described above. For consistent experimental conditions, all cell samples were imaged at comparable depths with the focal plane being ~ 300 nm away from the coverslip surface. The sample was concurrently illuminated with the 561 and 644 nm lasers each at ~ 2 kW/cm^2^, which led to the photoswitching of CF568 and AF647 single molecules. Fluorescence was recorded by the EM-CCD for a frame size of 256 × 256 pixels at 55 frames per second. Each movie was typically recorded for 20,000 frames.

### Antibody samples

For training of the dye-specific axial-localization ANNs of AF647 and CF568 single molecules, the above AF647- and CF568-labeled secondary antibodies were separately diluted in DPBS to ~ 2 µg/mL. Pre-cleaned #1.5 coverslips were separately incubated in either solution for ~ 5 min, briefly air-dried, rinsed with distilled water, and mounted and imaged as described above for cells. To record single-molecule images at different axial positions, the objective lens was scanned in the range of −700 to + 700 nm of the focal plane in 50 nm steps. Note that two separate set of scanned images are attained, each for AF647 and CF568. This enables independent neural network training for the two types of dyes, thus eliminating chromatic errors in the axial direction. Possible in-plane chromatic errors may further be corrected for through a bead calibration, but it has not been attempted in this work.

### Preprocessing of single-molecule images for ANNs

Single-molecule fluorescence in raw STORM and bead data was first identified and localized in 2D using established methods^1^. Here the goal was merely to obtain isolated single-molecule images as raw inputs of the ANNs, and similar results were obtained when using Insight3 (developed by Dr. Bo Huang at University of California, San Francisco and Dr. Xiaowei Zhuang at Harvard University) or ThunderSTORM^31^ (available at [https://github.com/zitmen/thunderstorm]) (Supplementary Fig. 5). Single-molecule PSF images were cropped as 13 × 13 pixels surrounding the 2D localizations. Here we rejected molecules that were too close to each other ( < 1 µm) and excluded abnormal single-molecule images with fitted widths (2*σ*) of > ~ 400 nm. The cropped PSF images were zero-centered, and their L2/Euclidean norm was normalized before being used as inputs for ANNs.

### Simulation of the PSF images

Realistic PSFs that account for the index discontinuity in the sample area were generated using the PSF generator package from EPFL^32^ ([http://bigwww.epfl.ch/algorithms/psfgenerator]) using the Gibson-Lanni (G-L) model^19^. Input parameters for the G-L model: NA = 1.45, immersion layer index = 1.51, sample layer index = 1.33, working distance = 130 μm, particle position = 1 μm. The emission wavelength was set to be 600 nm and 700 nm for two-color classification, and 700 nm for *z* estimation. For all experiments, PSF stack was firstly generated with 20 nm axial step size over ± 600 nm range and at 5 nm lateral resolution. For the final image, this high-resolution PSF stack was down-sampled into 100 nm pixel grid, and the total sum of the values within 24 × 24 pixel region-of-interest is matched to the given photon count and offset by the background photons. Lastly, detector shot-noise was modeled as the Poisson process with the rate matched to the mean photon counts within each pixel.

### Cubic-spline model-based maximum-likelihood estimation and classification

Openly available software from Zhuang group at Harvard University ([https://github.com/ZhuangLab/storm-analysis]^20^) was used to generate cubic-spline models for the simulated and experimental PSFs, calculate Cramer-Rao lower bounds (CRLBs)^27^, and MLE for *z* position. As the MLE *z* estimation for unmodified PSFs is prone to reaching local, rather than global minima^18^, two rounds of MLE fitting were performed with different initial *z* values ( + 300 nm/−300nm), and the one that yielded a lower likelihood error was selected. To perform MLE-based color classification, multiple error values, each from MLE fitting with one of the cubic spline models constructed with PSF stacks of the two different emission wavelengths, were returned and then compared to pick the color that finally minimizes the likelihood error. As for each color, MLE was done twice with different initial *z* values, four total rounds of MLE were thus required. For obtaining the PSF image stack for the spline models, while the ground truth of the 3D positions of the simulated PSFs are known by definition, for experiments on fluorescent beads ( ~ 15,000 photons), the in-plane positions were estimated through Gaussian least-squares fitting, and axial positions were from z scanning.

### Design and implementation of neural networks

An ANN architecture comprising multiple hidden layers was implemented using the Tensorflow framework on a computer with 32GB RAM, Intel i7-7800X CPU, and Nvidia GTX-1080Ti GPU. The same architecture was used for both the color-separating and axial-localization ANNs (4 total layers of 4096-4096-2048-1024 neurons, respectively; Supplementary Fig. 6). Each hidden layer was fully connected, and rectified linear units were used as their activation function. For color discrimination, Softmax function and cross-entropy were used for loss calculation, the weights in the network were not directly included for regularization, and a dropout layer was inserted before the final layer to prevent over-fitting. For axial/lateral localization, the output of the final layer was set to be a scalar value, and L2 norm was used to calculate the learning loss for each batch. In this case, L2 norm of the weights in each layer was added to the loss function for regularization, and dropout was not used. Network hyper-parameters such as the number of neurons in each layer (given above), the dropout ratio (0.5 for the color-separating ANN), and the regularization factor (0.01 for the axial-localization ANNs) were adjusted for optimized performance. The codes for our ANN implementation are available online ([https://github.com/ann-storm/ann-storm]).

### Neural network training

Since the network is subject to handling input images with various noise levels, it was essential to maintain a consistent noise level within the training dataset regardless of the classification class or axial location. Therefore, experimental PSF images with comparable photon counts of 4500-5500 were used throughout the training process, and molecules with photon counts higher than this range were used for the validation during the training process to check the generalization of the trained network. The weights initialized by the Xavier method^33^ are trained using the Adam optimization algorithm. An initial learning rate of 10^−4^ and 10^−3^, and a batch size of 64 and 32 were used for the color-separating and the axial-localization ANNs, respectively. The learning rate was set to decrease by ~ 5x after every 1,000 iterations. The sizes of the training sets were ~ 10,000 and ~ 6,000 per fluorophore type for the color-separating and the axial-localization ANNs, respectively. The networks converged within ~ 10 epochs (training time: 231.9 s for the color-separating ANN, and 488.5 s for the axial-localization ANNs).

### Neural network inference

At the inference stage, input single-molecule PSF images were first plugged into the color-separating ANN. This ANN provides a conditional probability distribution corresponding to the input image. Specifically, when the size of each input image is *N* by *N* pixels, and there are *M* different molecule color classes, the final output from the Softmax layer for the *i*th input image is:

1 $${\boldsymbol{P}}\left( {{\boldsymbol{y}}_{\boldsymbol{i}}{\mathrm{|}}{\boldsymbol{x}}_{\boldsymbol{i}}} \right),\,{\boldsymbol{x}}_{\boldsymbol{i}} \in {\boldsymbol{R}}^{{\boldsymbol{N}} \times {\boldsymbol{N}}},\,{\boldsymbol{y}}_{\boldsymbol{i}} \in \{ {\mathrm{Dye}}\,{\mathrm{1}},\, \cdots ,\,{\mathrm{Dye}}\,{\mathrm{M}}\} .$$

From this distribution, the ANN makes the decision in a maximum a posteriori (MAP) manner:^34^ through training, ANN provides the posterior distribution, and the molecule color class with the highest probability is chosen. This, in turn, implies that we can use this distribution to quantify the classification confidence. For example, in a simple binary classification problem, the confidence for the color assignment of the *i*th input image can be evaluated as:

2 $$\left| {{\mathbf{\Delta }}_{\boldsymbol{i}}} \right| = \left| {{\boldsymbol{P}}\left( {{\mathrm{Dye}}\,{\mathrm{1|}}{\boldsymbol{x}}_{\boldsymbol{i}}} \right) - {\boldsymbol{P}}\left( {{\mathrm{Dye}}\,{\mathrm{2|}}{\boldsymbol{x}}_{\boldsymbol{i}}} \right)} \right|.$$

By setting a finite confidence threshold *δ* to reject molecules with low classification confidences (*|Δ*_*i*_*|* < *δ*), improved classification accuracy may be obtained (Figs. 2i, j and 4j, k). This parameter may thus be adjusted by the user to balance the classification accuracy and rejection rate. Once the color of the molecule is determined, the single-molecule image is plugged into the axial-localization ANN trained for that particular color to evaluate the axial position. With GPU acceleration, both inference steps (passing the forward path of the neural networks) were extremely fast: only ~ 300 ms was used to infer 100,000 molecules for both the color-separating and the axial-localization ANNs.

### Reporting summary

Further information on research design is available in the Nature Research Reporting Summary linked to this article.

## Supplementary information

Supplementary Information

Reporting Summary

## Data Availability

STORM experiment training and evaluation data set are also available online on the code repository. Other data are available from the corresponding author upon reasonable request.

## Code Availability

The codes for color separation and axial localization ANNs are available online ([https://github.com/ann-storm/ann-storm], 10.5281/zenodo.2619228).

## References

[1] Rust MJ, Bates M, Zhuang X (2006). Sub-diffraction-limit imaging by stochastic optical reconstruction microscopy (STORM). Nat. Methods.

[2] Betzig E (2006). Imaging intracellular fluorescent proteins at nanometer resolution. Science.

[3] Hess ST, Girirajan TPK, Mason MD (2006). Ultra-high resolution imaging by fluorescence photoactivation localization microscopy. Biophys. J..

[4] Sharonov A, Hochstrasser RM (2006). Wide-field subdiffraction imaging by accumulated binding of diffusing probes. Proc. Natl Acad. Sci. USA.

[5] Yan R, Moon S, Kenny SJ, Xu K (2018). Spectrally resolved and functional super-resolution microscopy via ultrahigh-throughput single-molecule spectroscopy. Acc. Chem. Res..

[6] Moon S (2017). Spectrally resolved, functional super-resolution microscopy reveals nanoscale compositional heterogeneity in live-cell membranes. J. Am. Chem. Soc..

[7] Backlund MP, Lew MD, Backer AS, Sahl SJ, Moerner WE (2014). The role of molecular dipole orientation in single-molecule fluorescence microscopy and implications for super-resolution imaging. ChemPhysChem.

[8] von Diezmann A, Shechtman Y, Moerner WE (2017). Three-dimensional localization of single molecules for super-resolution imaging and single-particle tracking. Chem. Rev..

[9] Huang B, Wang W, Bates M, Zhuang X (2008). Three-dimensional super-resolution imaging by stochastic optical reconstruction microscopy. Science.

[10] Shtengel G (2009). Interferometric fluorescent super-resolution microscopy resolves 3D cellular ultrastructure. Proc. Natl Acad. Sci. USA.

[11] Bossi M (2008). Multicolor far-field fluorescence nanoscopy through isolated detection of distinct molecular species. Nano Lett..

[12] Zhang Z, Kenny SJ, Hauser M, Li W, Xu K (2015). Ultrahigh-throughput single-molecule spectroscopy and spectrally resolved super-resolution microscopy. Nat. Methods.

[13] Pavani SRP (2009). Three-dimensional, single-molecule fluorescence imaging beyond the diffraction limit by using a double-helix point spread function. Proc. Natl Acad. Sci. USA.

[14] Shechtman Y, Weiss LE, Backer AS, Lee MY, Moerner WE (2016). Multicolour localization microscopy by point-spread-function engineering. Nat. Photonics.

[15] Smith C, Huisman M, Siemons M, Grünwald D, Stallinga S (2016). Simultaneous measurement of emission color and 3D position of single molecules. Opt. Express.

[16] Siemons M, Hulleman CN, Thorsen ROslash, Smith CS, Stallinga S (2018). High precision wavefront control in point spread function engineering for single emitter localization. Opt. Express.

[17] Franke C, Sauer M, van de Linde S (2016). Photometry unlocks 3D information from 2D localization microscopy data. Nat. Methods.

[18] Li Y (2018). Real-time 3D single-molecule localization using experimental point spread functions. Nat. Methods.

[19] Gibson SF, Lanni F (1989). Diffraction by a circular aperture as a model for three-dimensional optical microscopy. J. Opt. Soc. Am. A.

[20] Babcock HP, Zhuang X (2017). Analyzing single molecule localization microscopy data using cubic splines. Sci. Rep..

[21] Kuntzer T, Tewes M, Courbin F (2016). Stellar classification from single-band imaging using machine learning. Astron. Astrophys..

[22] Newby JM, Schaefer AM, Lee PT, Forest MG, Lai SK (2018). Convolutional neural networks automate detection for tracking of submicron-scale particles in 2D and 3D. Proc. Natl Acad. Sci. USA.

[23] Zelger P (2018). Three-dimensional localization microscopy using deep learning. Opt. Express.

[24] Hershko E, Weiss LE, Michaeli T, Shechtman Y (2019). Multicolor localization microscopy and point-spread-function engineering by deep learning. Opt. Express.

[25] Bishop, C. M. *Neural Networks for Pattern Recognition*. (Oxford University Press, Inc., New York, NY, USA 1995).

[26] Zur RM, Jiang Y, Pesce LL, Drukker K (2009). Noise injection for training artificial neural networks: A comparison with weight decay and early stopping. Med. Phys..

[27] Liu S, Kromann EB, Krueger WD, Bewersdorf J, Lidke KA (2013). Three dimensional single molecule localization using a phase retrieved pupil function. Opt. Express.

[28] Hara, K., Vemulapalli, R. & Chellappa, R. Designing deep convolutional neural networks for continuous object orientation estimation. Preprint at https://arxiv.org/abs/1702.01499 (2017).

[29] Dempsey GT, Vaughan JC, Chen KH, Bates M, Zhuang X (2011). Evaluation of fluorophores for optimal performance in localization-based super-resolution imaging. Nat. Methods.

[30] Cabriel C, Bourg N, Dupuis G, Lévêque-Fort S (2018). Aberration-accounting calibration for 3D single-molecule localization microscopy. Opt. Lett..

[31] Ovesný M, Křížek P, Borkovec J, Švindrych Z, Hagen GM (2014). ThunderSTORM: a comprehensive ImageJ plug-in for PALM and STORM data analysis and super-resolution imaging. Bioinformatics.

[32] Kirshner H, Aguet F, Sage D, Unser M (2013). 3-D PSF fitting for fluorescence microscopy: implementation and localization application. J. Microsc..

[33] Glorot, X. & Bengio, Y. in *Proceedings of the Thirteenth International Conference on Artificial Intelligence and Statistics,* 249–256 (2010).

[34] Richard MD, Lippman RP (1991). Neural network classifiers estimate Bayesian a posteriori probabilities. Neural Comput..

