# Supplementary material for: Information-rich localization microscopy through machine learning
Source: Nat Commun. 2019 Apr 30;10:1996. doi: 10.1038/s41467-019-10036-z (PMC6491467; doi:10.1038/s41467-019-10036-z)
Supplement: Supplementary file 1 — Supplementary Information [file 41467_2019_10036_MOESM1_ESM.pdf]

## Supplementary Information

# Information-rich localization microscopy through machine learning

Taehwan Kim[1†], Seonah Moon[2†], & Ke Xu[2,3*]

[1]*Department of Electrical Engineering and Computer Sciences, University of California, Berkeley, CA 94720*

[2]*Department of Chemistry, University of California, Berkeley, CA 94720*

[3]*Chan Zuckerberg Biohub, San Francisco, CA 94158*

[†]*These authors contributed equally*

**Corresponding author (email: xuk@berkeley.edu)*

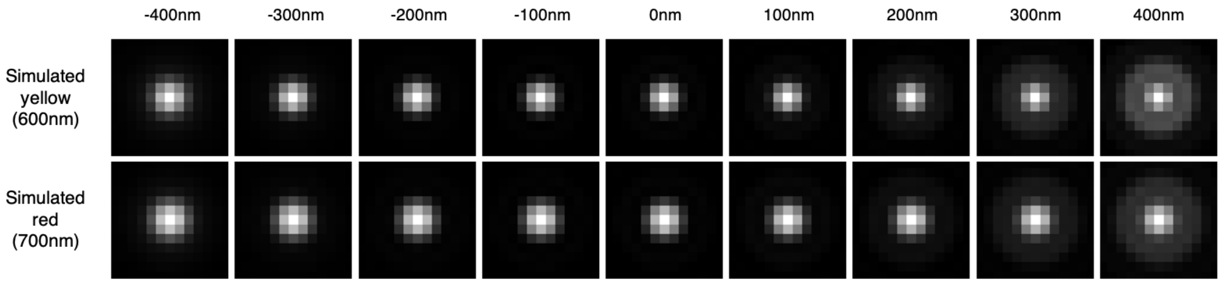


**Supplementary Figure 1.** Simulated PSFs (Gibson-Lanni model) for emission wavelengths of 600 nm (yellow) and 700 nm (red) at different axial positions.

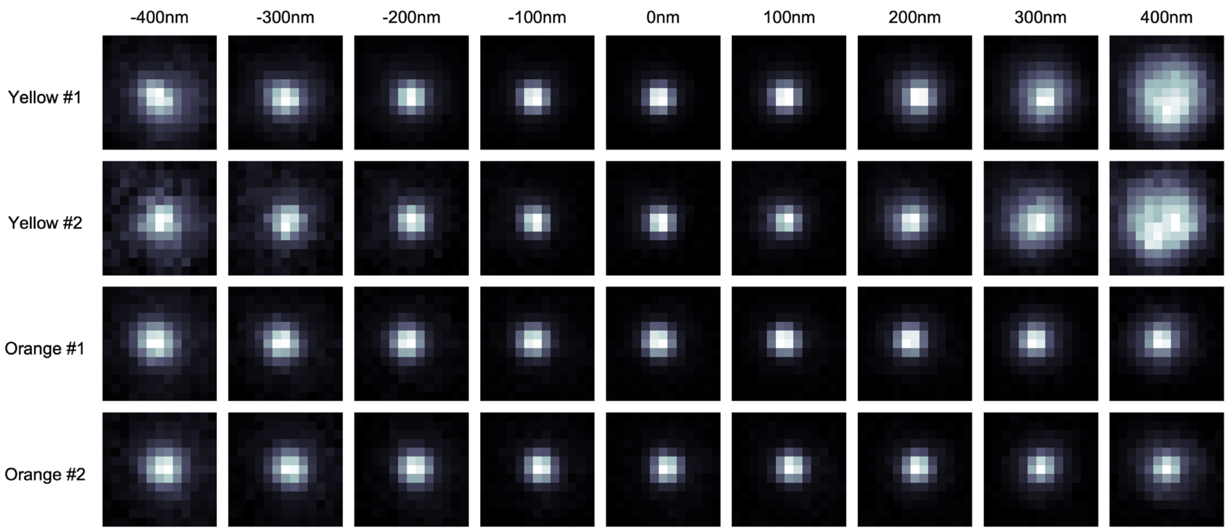


**Supplementary Figure 2.** Experimental images of two types (yellow/orange) of fluorescent beads taken at different axial positions. Two examples are shown at each z-position for each color, to illustrate complications due to different pixelation effects.

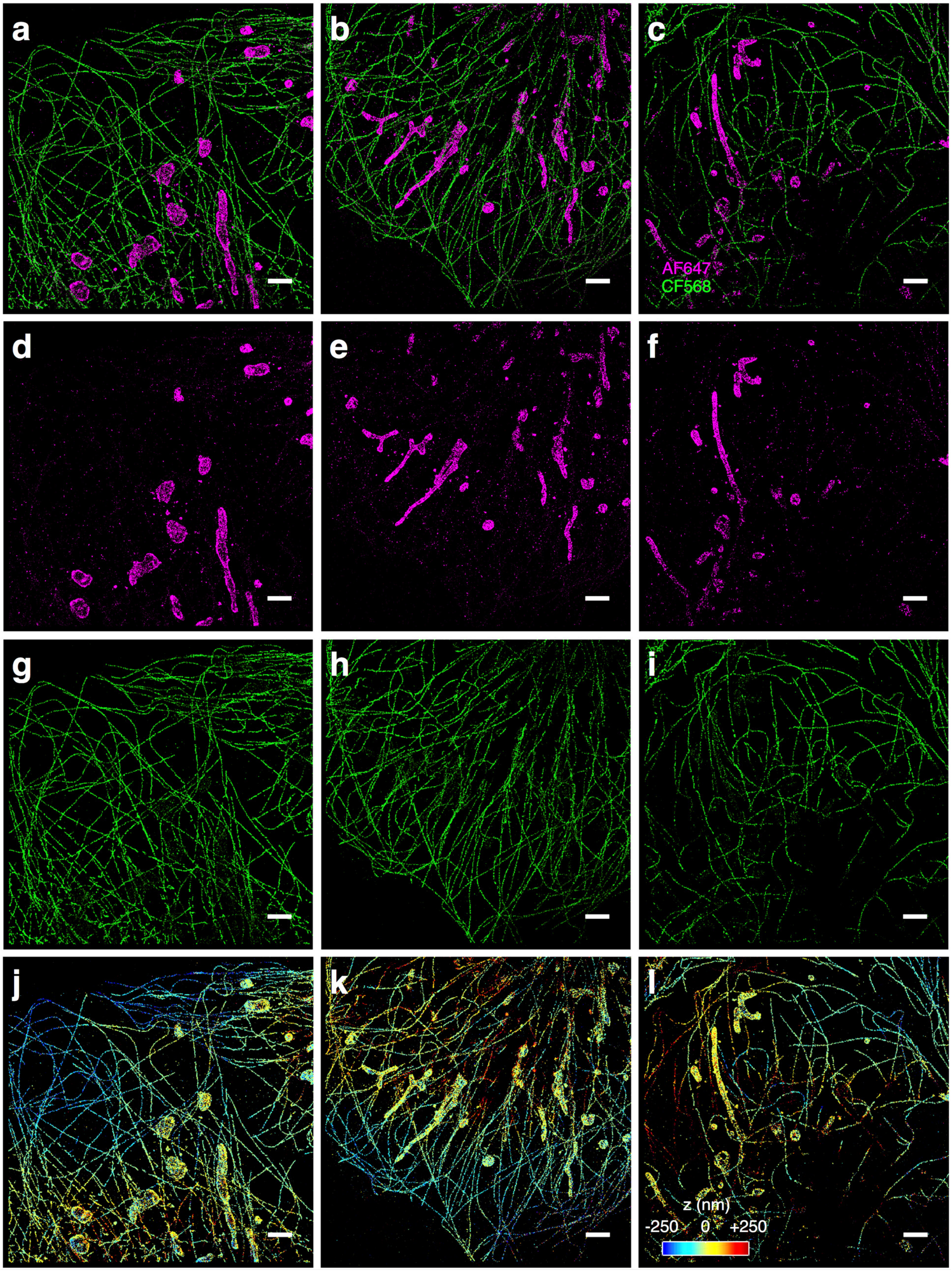


**Supplementary Fig. 3.** Additional examples of ANN-resolved multicolor 3D SMLM of fixed cells based on unmodified PSFs. Scale bars, 2 µm.

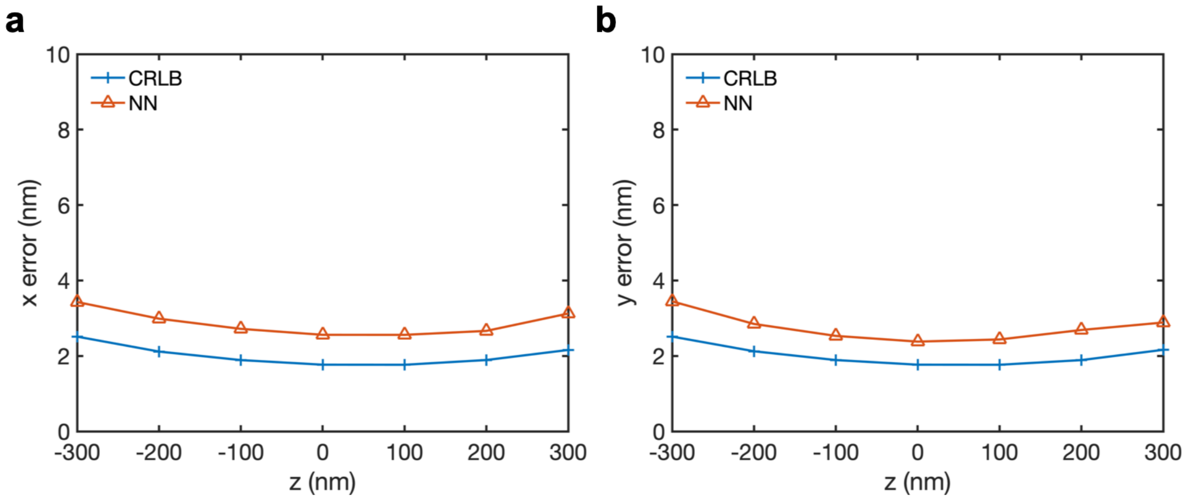


**Supplementary Figure 4.** Localization precisions of the ANN-based estimation of (**a**) *x* and (**b**) *y* for simulated PSFs (5000 photons, 10 background photons/pixel, *z*=0), compared with the Cramer-Lao lower bound calculated from the cubic-spline model.

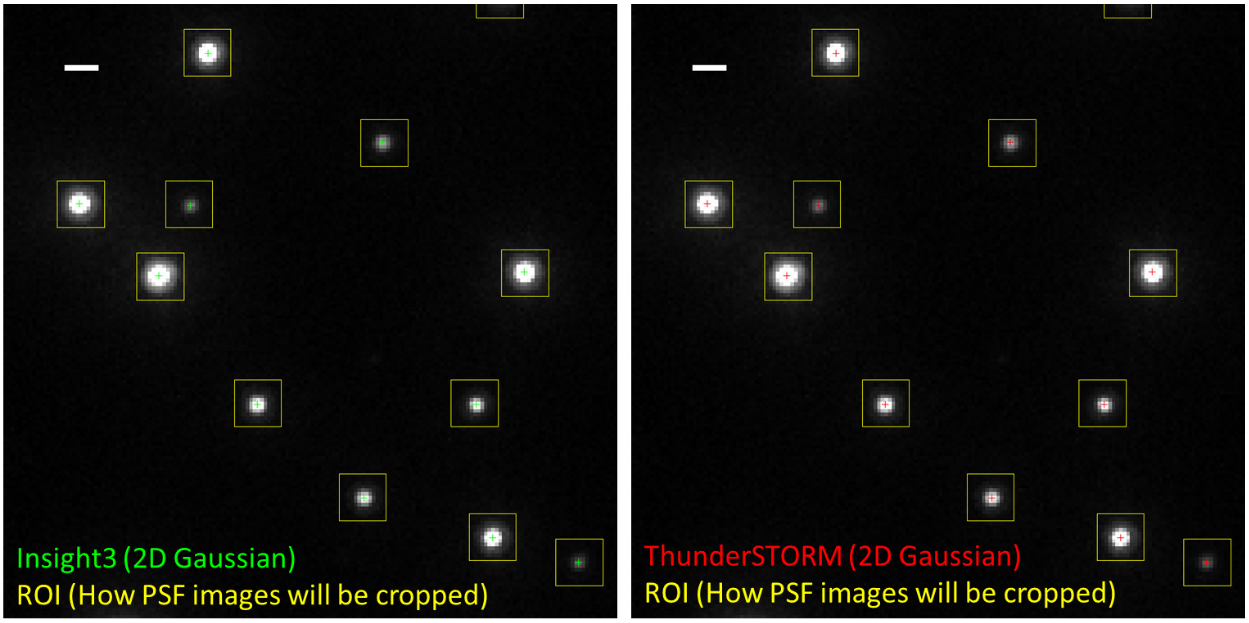


**Supplementary Figure 5.** Detected region-of-interests (ROIs; yellow boxes) of 13×13 pixels for the experimental PSFs of "orange" fluorescent beads from two different SMLM software, Insight3 (left) and ThunderSTORM (right). Scale bars, 1 µm

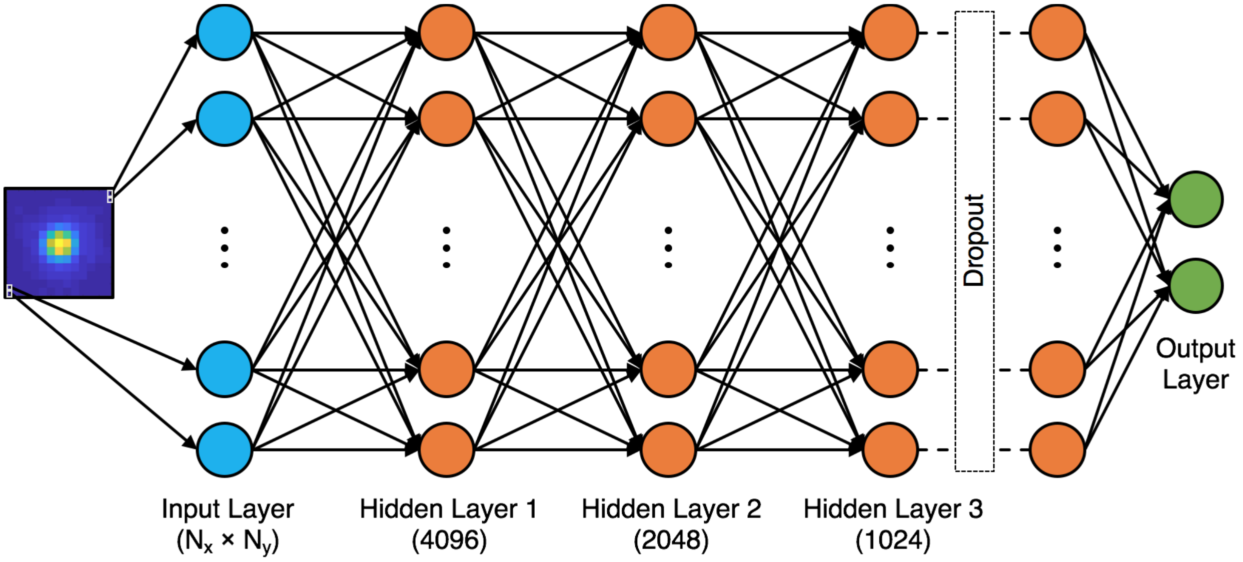


**Supplementary Figure 6.** Architecture of the artificial neural network used in the paper.
